# Sensitive and Compact Evanescent-Waveguide Optical Detector for Sugar Sensing in Commercial Beverages

**DOI:** 10.3390/s23198184

**Published:** 2023-09-30

**Authors:** Alessio Buzzin, Rita Asquini, Domenico Caputo, Giampiero de Cesare

**Affiliations:** Department of Information Engineering, Electronics and Telecommunications, Sapienza University of Rome, via Eudossiana 18, 00184 Rome, Italy; rita.asquini@uniroma1.it (R.A.); domenico.caputo@uniroma1.it (D.C.); giampiero.decesare@uniroma1.it (G.d.C.)

**Keywords:** lab-on-chip, optical biosensor, thin-film photosensor, polymer waveguide, sugar, commercial beverages

## Abstract

This work presents a compact and sensitive refractive index sensor able to evaluate the concentration of an analyte in a sample. Its working principle leverages on the changes in the optical absorption features introduced by the sample itself on the evanescent waves of a light beam. The device’s high compactness is achieved by embedding the sample–light interaction site and the detector in a 1 cm^2^ glass substrate, thanks to microelectronics technologies. High sensitivity is obtained by employing a low-noise p-i-n hydrogenated amorphous silicon junction, whose manufacture process requires only four UV lithographic steps on a glass substrate, thus ensuring low production costs. The system’s capabilities are investigated by sensing the sugar content in three commercial beverages. Sensitivities of 32, 53 and 80 pA/% and limits of detection of 47, 29 and 18 ppm are achieved. The above performance is comparable with state-of-the-art results available in the literature, where more complex optical setups, expensive instrumentation and bulky devices are used.

## 1. Introduction

During recent decades, tackling obesity has become one of the most critical challenges for global health, as the incidence of overweight people among the world population has been growing at increasing rates [1]. Being overweight is the primary risk factor in a variety of diseases, such as diabetes, heart-related pathologies and many forms of cancer [2,3]. Although the causes of obesity can be found in unhealthy lifestyles, unregulated hormone production or bad diet habits [4], the consumption of sugar-sweetened beverages plays an important role, as supported by strong and established scientific data [5,6]. National and international organizations have been increasingly concerned about this phenomenon and its effects on global health and national health-care costs. To address these issues, dietary guidelines and policies have been released, recommending upper limits on calorie intake from sugars, promoting health education campaigns and implementing excises on sugar-sweetened beverages [7]. On the one hand, this approach positively affected the general population’s behavior in purchasing these drinks; on the other hand, it stimulated fraud, drink reformulations, and price and marketing strategies [8]. Moreover, foods’ nutritional labels are still not mandatory in many countries; nonetheless, they can be manipulated to influence the consumer’s perception [9,10].

The worldwide growing attention on human health and food quality control is increasingly pushing both industry and research to develop new, smarter and less expensive sensing procedures [11]; impedance, amperometric and electrochemical methods [12,13] are being replaced by optical techniques such as refractive index sensing, due to their sensitivity and specificity [14]. However, these techniques often suffer from complex sample pre-treatment, high costs and a lack of compactness due to the need of off-chip instrumentation [15]. For example, Azargoshasb et al. [16] developed an evanescent waveguide sensor, reporting good performances for sensitivity and limit of detection (LoD) in similar applications. However, the system still relies on distinct modules, and the detection phase is performed using off-chip expensive instrumentation. Moreover, the chip itself consists of a section of optical fiber whose core has been treated with complex and expensive procedures.

To overcome these drawbacks, we report on a compact and sensitive refractive index sensor, based on the evanescent wave absorbance phenomenon. Operations need neither sample pre-treatment nor complex optics, thanks to an embedded, thin-film photodetector. Recently, we successfully reported the system’s achievements in blood analysis [17] and milk fat content detection [18]. Here, we present a performance analysis for the detection of sugar concentration in commercial carbonated beverages, taking advantage of the linear relationship between the refractive index of sugar-sweetened drinks and their sugar content, which has been extensively proven in the past [19,20].

## 2. Operating Principle and Features

**Figure 1:**
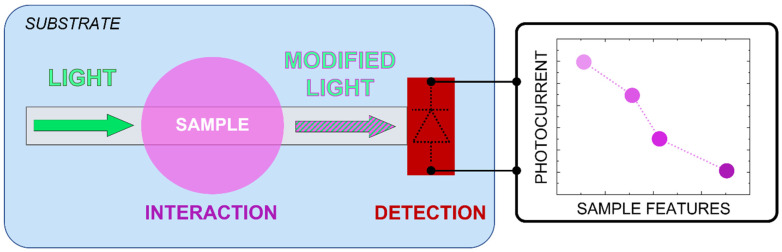
Operating principle of the presented system: the sensor promotes light absorption in the sample under investigation due to the evanescent wave phenomenon and monitors light variations (proportional to the amount of analyte inside the sample) with the embedded photodetector. The graph on the right is a qualitative representation of the photocurrent change with the sample optical characteristics.

The proposed system is sketched in Figure 1. The device integrates a borosilicate glass substrate (Borofloat33 by SCHOTT), SU-8 (3005 series by Microchem) polymer and an amorphous silicon (a-Si:H) p-i-n stacked structure. The SU-8 acts as an optical waveguide to convey light while hosting a sample on its top surface. This sample-waveguide “interaction” area, as referred to in Figure 1, is designed to allow the dispersion of part of the light’s evanescent wave into the sample, which absorbs part of the light according to its complex refractive index. As a consequence, the light power detected by the a-Si:H photodiode at the waveguide’s output can provide information on the amount of the analyte if the sample’s optical properties depend on the concentration of such an analyte [21].

### 2.1. Optical Waveguide

As waveguiding material, the SU-8 polymer (3005 series, by Microchem) represents a valid choice, due to its low propagation losses in the visible spectrum [22,23]. Plus, being a photosensitive polymer, SU-8 is quick and easy to pattern using a standard UV-lithography process [22,24]. Indeed, SU-8 has a well-known history in a variety of microelectronics, MEMS and optical applications [25,26,27,28]. For these reasons, the SU-8 polymer (refractive index in the green spectrum equal to 1.58, according to “SU-8 3000 data sheet” by MICROCHEM) is the optical channel core, while the glass substrate (refractive index in the green spectrum equal to 1.48, according to “SCHOTT optical glasses data sheets”) acts as lower cladding. Air is used as upper cladding.

### 2.2. Optical Detector

To monitor the output light at the end of the sample-waveguide interaction area, a p-doped/intrinsic/n-doped hydrogenated amorphous silicon (a-Si:H) photodiode is used. Over the years, a-Si:H has been employed as a photoresponsive semiconductor in a wide range of applications that combine large area and low cost, such as sensing [29,30], photovoltaics [31], MEMS [30] and integrated photonics [32,33]. Its success stems from the ability to fabricate p-i-n junctions with hydrogen-saturated dangling bonds, by using plasma-enhanced chemical vapor deposition (PECVD) technology [34]. This technique enables the maximization of electro-optical responsivity and high quantum efficiency, and the minimization of shot noise current (around 1 fA/$\sqrt{\mathrm{Hz}}$) [35].

In such structures, the intrinsic layer is the active region of the device and its thickness allows the selection of the detection window of the photodiodes [34]. For the purpose of targeting visible light, a 500 nm thick intrinsic layer is designed.

To connect the p-doped and n-doped layer to the external circuitry, two metallization levels are needed, with an intermediate insulation layer. To protect the junction and, at the same time, to expose it to the sensor’s guided light, the photodiode is buried inside the waveguide; in this configuration, the SU-8 polymer acts both as an optical channel and electrical insulator, minimizing the number of photolithographic masks required for the device’s fabrication. Moreover, such a design has already been adopted in the past, with an experimentally demonstrated coupling of light from an SU-8 waveguide to a silicon photodiode [18,36].

**Figure 2:**
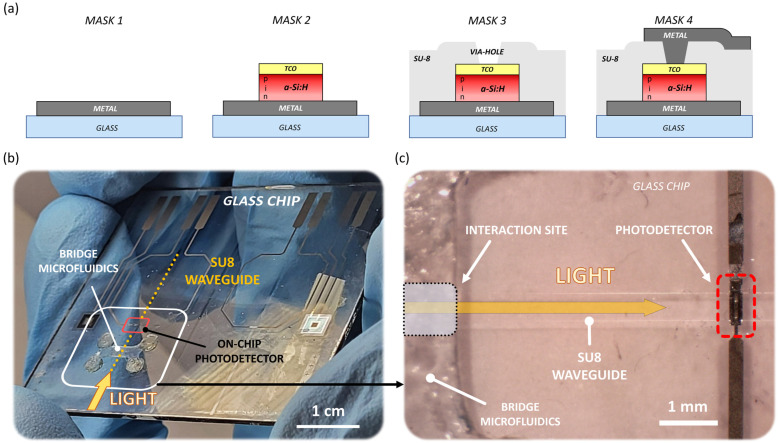
(**a**) Lithographic steps for the fabrication procedure: definition of the bottom n-side contact of the detector (“MASK 1”); patterning of the a-Si:H n-i-p stacked photojunction and its ITO p-side top contact (“MASK 2”); shaping of the SU-8 optical waveguide and, concurrently, the detector’s electrical insulation, with via-holes (“MASK 3”); definition of the top metal connection from the via-holes to the edge of the substrate (“MASK 4”). (**b**) Fabricated prototype. (**c**) Microscope picture of the system.

While metals are used as the junction’s bottom contact and interconnections, a thin film of indium-tin oxide (ITO) is chosen as junction’s top contact. ITO is a transparent conductive oxide (TCO) often employed in operations requiring electrical conductivity and optical transparency, such as displays, photovoltaics and optoelectronic devices [37,38]. ITO has a high refractive index in the visible spectrum, reaching a value of 1.88 in the green range [39]; this enables its use as optical buffer between the waveguide and the diode to facilitate the coupling of light. Moreover, the electrical conductivity of ITO enables its use as the diode’s p-side electrode to collect the photogenerated charges and to connect the diode to the external circuitry [40]. A schematic cross-section of the device is shown in Figure 2a (“MASK 4”).

## 3. Prototyping

The proposed sensor is obtained by monolithically integrating the a-Si:H photodiode and the polymer waveguide on glass [17,41]. The manufacturing procedure involves thin-film microelectronic technologies and consists of four lithographic steps, as reported in Figure 2a. The first mask models the bottom n-side contact of the detector and the electrical connection to the substrate’s edge. As bottom metal, a chromium/aluminum/chromium (Cr/Al/Cr) sandwich is deposited through vacuum evaporation; Al is chosen for its high electrical conductivity, whereas Cr is used for its excellent adhesion to glass and as a protective layer to avoid Al oxidation. The second mask shapes the a-Si:H n-i-p stacked photojunction and its ITO p-side top contact. The thicknesses of the n-i-p layers are equal to 50 nm/500 nm/10 nm, respectively. The PECVD process is executed under 200 °C, with a 25 mW/cm^2^ input power. RF magnetron sputtering is used to obtain the ITO film; the deposition is performed at 200 °C and with an input power equal to 1.1 W/cm^2^, achieving a thickness equal to 120 nm. The third mask molds the SU-8 optical waveguide and, concurrently, the detector’s electrical insulation, with via-holes. Finally, the fourth mask models the top metal connection from the via-holes to the edge of the substrate. The top metal is obtained by sputtering a 250 nm thick film of a titanium–tungsten alloy. Figure 2b shows a (5 × 5) cm^2^ glass substrate after the fabrication procedure. The white inset in Figure 2b includes the entire device, which covers an overall footprint of about 1 cm^2^. This inset is enlarged in the microscope picture in Figure 2c, which shows part of the interaction site, the SU-8 waveguide and the embedded detector. The system also includes a microfluidic bridge-like structure, consisting of razor-cut, plastic-made pressure-sensitive adhesives, designed and tested [18] with the specific purpose of delivering the sample to the optical waveguide without interfering with the evanescent-wave interaction.

**Figure 3:**
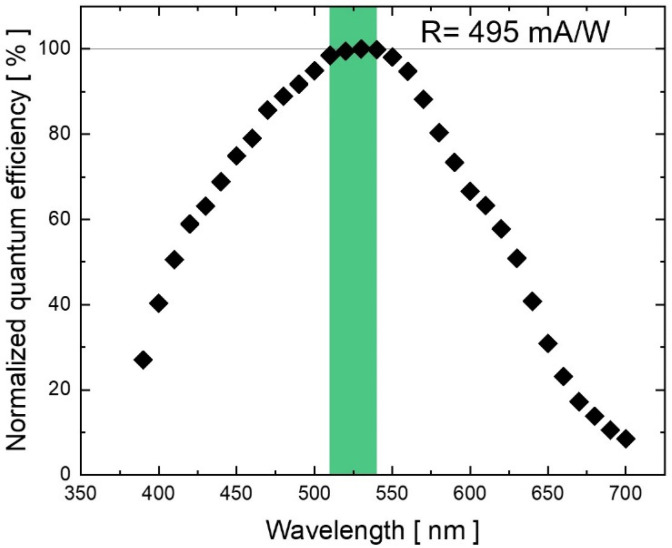
Normalized quantum efficiency of the fabricated detector vs. wavelength in the visible spectrum. At the photodiode’s photoresponse peak (around 530 nm), the measured quantum electro-optical responsivity is equal to 495 mA/W.

To check the photodiode’s characteristics, we measure its quantum efficiency (QE) in the visible spectrum. A monochromator (SPEX 340 from Jobin Yvon) is used to select light beams between 400 nm and 700 nm of wavelength coming from a halogen lamp. The amount of optical power hitting the photodiode is then compared to the generated photocurrent at each wavelength in a calibrated crystalline silicon photodiode (DR 2550 from EG&G Gamma Scientific, San Diego, CA, USA). The result is a QE peak around 530 nm. At this wavelength, a diode’s photocurrent of 4.95 mA is registered for a 10 mW impinging light beam, leading to an electro-optical responsivity (R) value equal to 495 mA/W. Figure 3 plots the normalized quantum efficiency of the device with respect to the working wavelength.

## 4. Sensing Demonstration of Sugar Content in Commercial Beverages

Yusmawati et al. [42] characterized the dielectric properties of three commercial beverages (“F&N Orange”, “100 Plus” and “Coke”). In their study, the authors determined the real (ε_r_) and imaginary (ε_i_) parts of the dielectric constant of the beverage solutions with sugar contents up to 12%, using surface plasmon resonance (SPR) and a 632.8 nm He-Ne laser source. Starting from these data, our work focuses on converting the dielectric permittivity to refractive index. At optical frequencies, the real and imaginary parts of the complex dielectric permittivity are related to the real (n) and imaginary (k) parts of the complex refractive index through the following equations, respectively:

(1) $$\varepsilon_{r}=n^{2}-k^{2}$$

(2) $$\varepsilon_{i}=2\mathrm{nk}$$

(3) $$n^{2}=\frac{\varepsilon_{r}}{2}+\frac{1}{2}\sqrt{\varepsilon_{r}^{2}+\varepsilon_{i}^{2}}$$

(4) $$k=\frac{\varepsilon_{i}}{2n}$$

where k is commonly referred to as the extinction coefficient of the medium and determines the amount of optical absorption inside that medium [43]. The result of our conversion is reported in Table 1.

**Figure 4:**
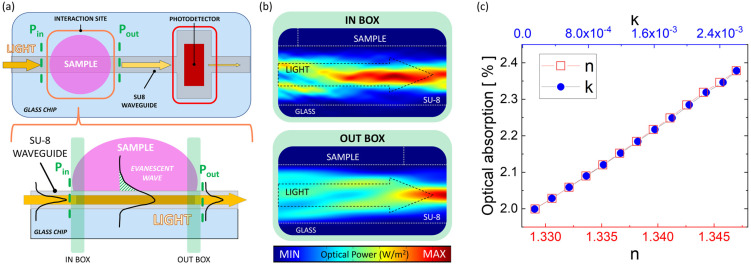
(**a**) Top view of the sensor (top) and side view of the interaction site (bottom). (**b**) Evolution of the optical power flowing (from left to right) before (“IN BOX”) and after (“OUT BOX”) the transit below the sample. (**c**) Simulated optical absorption in the interaction site when the sample is modeled as the “F&N Orange” solutions (see Table 1). The simulations are performed at 632.8 nm at different values of the refractive index’s real (n) and imaginary (k) part, corresponding to different beverage concentrations in water.

The obtained values of n and k, reported in Table 1, are used to model the optical properties of samples with different sugar concentrations. The interaction with the guided light’s evanescent wave is evaluated by calculating the portion of the light absorbed by the sample; this absorption changes the optical power exiting the interaction site. COMSOL Multiphysics is used to model the longitudinal section of the interaction site with the optical features of the materials. Figure 4a depicts a schematic of the system’s layout: a general top-view is shown on the top, while a side-view of the interaction site (as modeled in COMSOL) is depicted on the bottom. Maxwell’s equations are implemented (through the “electromagnetic waves: frequency domain” physics module) and used to evaluate the optical power flowing through the input (P_in_) and output (P_out_) of the model, with a light excitation at 632 nm wavelength. The normalized optical absorption descends from (P_in_-P_out_)/P_in_. Figure 4b shows the spatial evolution of the optical power flowing along the SU-8 waveguide before (“IN BOX” on the top) and after (“OUT BOX” on the bottom) the transit below the sample. Figure 4c plots the simulated absorbed optical power with respect to the real part (n) and imaginary part (k) of the “F&N Orange” solutions when the sample-waveguide overlapping length is equal to 300 μm. The behavior is linear, and spans from 2% absorption when n equals 1.33 and k equals 3.57 × 10^−4^, to nearly 2.4% absorption when n is 1.347 and k is 2.6 × 10^−3^.

**Figure 5:**
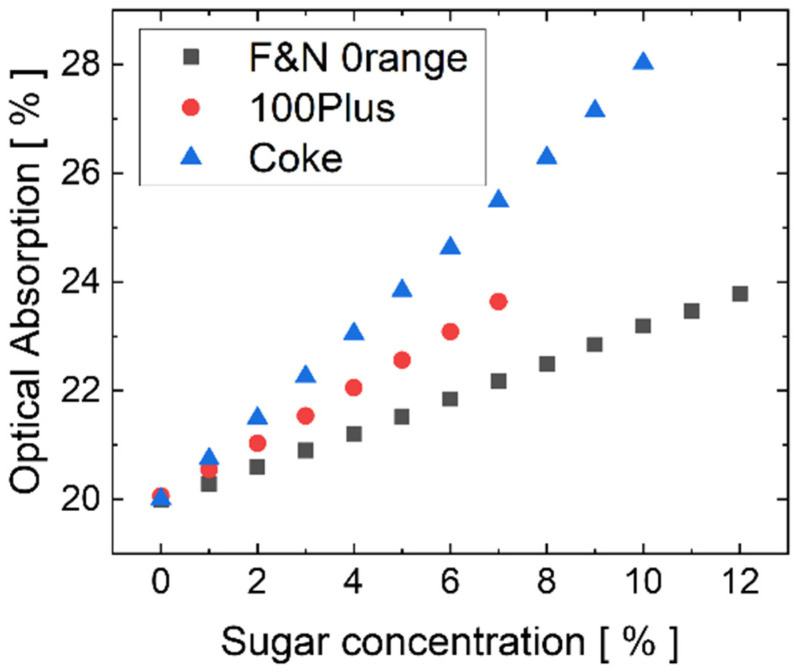
(Simulation results of the evanescent-wave optical absorption vs. sugar concentration ((P_in_−P_out_)/P_in_) for the 3 commercial beverages [42] at different sugar concentrations. Values of n and k reported in Table 1 have been used for the purpose.

Based on these preliminary results, a simulation campaign is carried out considering the fabricated layout, with a 3 mm long interaction site. Figure 5 plots the calculated optical absorption due to the interaction with the three modeled solutions (as reported in Table 1). The behavior is linear, with an optical absorption spanning from 20% without sugar, up to 28% in the case of “Coke” with 10% sugar content. Compared with Figure 4c, the increase in the interaction length causes a higher optical absorption, which, in turn, leads to a larger variation in the output power and then to a larger variation in the a-Si:H photocurrent. This achievement is desired because, as shown below, an increased photocurrent variation with sugar concentration determines a better LoD. On the other hand, too long an interaction site length can cause a complete absorption of the guided light and then too low a light intensity absorbed by the photodiode. Therefore, a properly designed interaction site length is necessary to achieve optimal system performance. In this regard, it must be noted that, to avoid a complete optical absorption in the interaction site and, consequently, the system’s failure, such an issue can be addressed in the design phase by carrying out an extensive simulation campaign, in which the length of the interaction site is progressively increased until the output optical power goes to zero (i.e., total absorption and sensing failure). The length of the interaction site must be set within the boundaries given by the results of the simulations. The lower boundary corresponds to a short interaction site, where the optical absorption is too low to have a good sensing performance. This occurs when the changes in output optical power are comparable to the natural fluctuations in the optical power of the light source or are comparable to the Schottky noise current contribution of the thin-film a-Si:H detector. The higher boundary corresponds to a long interaction site, with 100% optical absorption, and to the failure of the system. The subsequent system’s development steps (design of the lithographic masks, fabrication) must then be carried out with these results as design rules.

**Figure 6:**
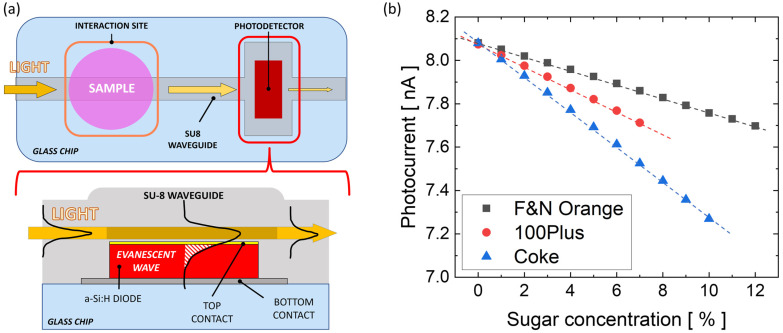
(**a**) Top view of the sensor (**top**) and side view of the detection site (**bottom**). (**b**) Estimated photocurrent vs. sugar concentration for the 3 commercial beverages [42].

As depicted in Figure 6a, the guided light comes out of the interaction site and then is coupled in the detector through evanescent-wave absorption. To evaluate the performance of the whole system, the data collected from the COMSOL simulations are combined with the experimental data gathered from the electro-optical characterization of the fabricated photodiode. In our assessment, the optical power hitting the detector is assumed to be 40 nW before placing the sample over the waveguide. Considering this specific incident power as a starting condition, and taking into account that the experimentally measured diode’s responsivity at 633 nm is 252 mA/W, equal to 50.9% with respect to the peak at 530 nm [18] (495 mA/W, see Figure 3), the resulting detector’s starting photocurrent is 10.1 nA.

Figure 6b reports the detector’s estimated photocurrents when placing the three beverages at different sugar concentrations. As a result, the estimated sensitivity of the sensor is about 32 pA/%, 53 pA/% and 80 pA/% for “F&N Orange”, “100 Plus” and “Coke” [42], respectively.

Under these operating conditions, with 10.1 nA being the highest possible current flowing through the p-i-n junction, the detector’s Schottky noise contribution is 56.8 fA (considering that the operating bandwidth in our case is 1 Hz) in the worst possible scenario. The minimum detectable signal (MDS) can be derived as three times the Schottky noise and is equal to 170.4 fA. The LoD can be evaluated as the MDS divided by the sensitivity. In this scenario, the LoD is 47 ppm, 29 ppm and 18 ppm for sugar concentration in “F&N Orange”, “100 Plus” and “Coke” solutions, respectively.

## 5. Conclusions

This work presents the evaluation of the performance of a compact and low-cost evanescent-waveguide optical sensor capable of detecting the concentration of an analyte inside a sample by responding to changes in its complex refractive index. The dielectric properties of three commercial beverages, found in the literature and converted to refractive indexes and extinction coefficients, are used to investigate the sensing capabilities of the proposed system. Optical simulations are performed using COMSOL Multiphysics to evaluate the drops in the power guided inside the system’s optical channel due to the evanescent wave interaction with the modeled samples. The calculated data are combined with the experimental values of the photodiode response to infer the whole system’s performance. The results in terms of sugar sensitivities span from 32 to 80 pA/%, depending on the soft drink; likewise, the expected LoD ranges from 47 to 18 ppm. These values are better than those achieved using optical analyzers (such as digital refractometers) currently available on the market for similar applications. Moreover, the results are comparable with state-of-the-art data reported in the literature, where more complex optical setups, expensive instrumentation and bulky devices are employed [14]. Future works include the arrangement of an experimental campaign to confirm this investigation. Tests will be performed with multiple interaction site lengths to confirm what comes from the simulations, and to experimentally achieve the failure of the sensing system. Experiments will also be conducted at different wavelengths (inside the visible spectrum), to obtain a more complete optical characterization of the chosen commercial beverages, also focusing on the repeatability of the measurements. In this regard, the interchangeability of the light sources can be seen as one of the most valuable assets of our device, providing great versatility; as long as the working wavelength is in the detection window of the thin-film a-Si:H photodetector, light sources can be changed and the wavelength can be tuned according to the specific analyte under investigation. In addition to this, it should be noted that the developed system can be upgraded as an ultra-compact, integrated optical interferometer [44,45] by simply changing the geometry of the waveguide; a preliminary feasibility study in this direction has been recently published by our research group [46]. This may play an important role in the field of optical interferometry for biosensing, as it improves the compactness of the system, making it more suitable for in-field scenarios.

## Figures and Tables

**Table 1:** Real (n) and imaginary (k) values of the refractive index of three commercial drinks at different sugar concentrations (c) as achieved from the dielectric properties found in [42].

	N			k		
Sugar [%]	F&N Orange	100 Plus	Coke	F&N Orange	100 Plus	Coke
0	1.329	1.329	1.329	1.50 × 10^−4^	1.99 × 10^−4^	1.59 × 10^−4^
1	1.330	1.330	1.331	3.57 × 10^−4^	5.40 × 10^−4^	6.80 × 10^−4^
2	1.332	1.332	1.333	5.64 × 10^−4^	8.80 × 10^−4^	1.20 × 10^−3^
3	1.333	1.334	1.335	7.70 × 10^−4^	1.22 × 10^−3^	1.72 × 10^−3^
4	1.335	1.335	1.336	9.76 × 10^−4^	1.56 × 10^−3^	2.24 × 10^−3^
5	1.336	1.337	1.338	1.18 × 10^−3^	1.90 × 10^−3^	2.75 × 10^−3^
6	1.338	1.339	1.340	1.39 × 10^−3^	2.23 × 10^−3^	3.27 × 10^−3^
7	1.339	1.340	1.342	1.59 × 10^−3^	2.57 × 10^−3^	3.78 × 10^−3^
8	1.341		1.344	1.79 × 10^−3^		4.29 × 10^−3^
9	1.342		1.346	2.00 × 10^−3^		4.80 × 10^−3^
10	1.344		1.348	2.20 × 10^−3^		5.31 × 10^−3^
11	1.346			2.40 × 10^−3^		
12	1.347			2.60 × 10^−3^		

## Data Availability

Not applicable.

## References

[1] Bentham J., Di Cesare M., Bilano V., Bixby H., Zhou B., Stevens G.A., Riley L.M., Taddei C., Hajifathalian K., Lu Y. (2017). Worldwide trends in body-mass index, underweight, overweight, and obesity from 1975 to 2016: A pooled analysis of 2416 population-based measurement studies in 128 9 million children, adolescents, and adults. Lancet.

[2] GBD 2015 Obesity Collaborators (2017). Health Effects of Overweight and Obesity in 195 Countries over 25 Years. N. Engl. J. Med..

[3] Malik V.S., Hu F.B. (2022). The role of sugar-sweetened beverages in the global epidemics of obesity and chronic diseases. Nat. Rev. Endocrinol..

[4] Mozaffarian D., Hao T., Rimm E.B., Willett W.C., Hu F.B. (2011). Changes in Diet and Lifestyle and Long-Term Weight Gain in Women and Men. N. Engl. J. Med..

[5] Malik V.S., Popkin B.M., Bray G.A., Després J.P., Hu F.B. (2010). Sugar-sweetened beverages, obesity, type 2 diabetes mellitus, and cardiovascular disease risk. Circulation.

[6] Malik V.S., Schulze M.B., Hu F.B. (2006). Intake of sugar-sweetened beverages and weight gain: A systematic review. Am. J. Clin. Nutr..

[7] Scarborough P., Adhikari V., Harrington R.A., Elhussein A., Briggs A., Rayner M., Adams J., Cummins S., Penney T., White M. (2020). Impact of the announcement and implementation of the UK soft drinks industry levy on sugar content, price, product size and number of available soft drinks in the UK, 2015–2019: A controlled interrupted time series analysis. PLoS Med..

[8] Silver L.D., Ng S.W., Ryan-Ibarra S., Taillie L.S., Induni M., Miles D.R., Poti J.M., Popkin B.M. (2017). Changes in prices, sales, consumer spending, and beverage consumption one year after a tax on sugar-sweetened beverages in Berkeley, California, US: A before-and-after study. PLoS Med..

[9] Muller L., Ruffieux B. (2020). What makes a front-of-pack nutritional labelling system effective: The impact of key design components on food purchases. Nutrients.

[10] Ducrot P., Julia C., Méjean C., Kesse-Guyot E., Touvier M., Fezeu L.K., Hercberg S., Péneau S. (2016). Impact of different front-of-pack nutrition labels on consumer purchasing intentions: A randomized controlled trial. Am. J. Prev. Med..

[11] Kaushik A., Mujawar M.A. (2018). Point of care sensing devices: Better care for everyone. Sensors.

[12] Daikuzono C.M., Delaney C., Morrin A., Diamond D., Florea L., Oliveira O.N. (2019). Paper based electronic tongue-a low-cost solution for the distinction of sugar type and apple juice brand. Analyst.

[13] Salman F., Kazici H.C., Kivrak H. (2020). Electrochemical sensor investigation of carbon-supported PdCoAg multimetal catalysts using sugar-containing beverages. Front. Chem. Sci. Eng..

[14] Belay A., Assefa G. (2018). Concentration, Wavelength and Temperature Dependent Refractive Index of Sugar Solutions and Methods of Determination Contents of Sugar in Soft Drink Beverages using Laser Lights. J. Lasers Opt. Photonics.

[15] Svanberg S., Zhao G., Zhang H., Huang J., Lian M., Li T., Zhu S., Li Y., Duan Z., Lin H. (2016). Laser spectroscopy applied to environmental, ecological, food safety, and biomedical research. Opt. Express.

[16] Azargoshasb T., Navid H.A., Parvizi R., Heidari H. (2020). Evanescent Wave Optical Trapping and Sensing on Polymer Optical Fibers for Ultra-Trace Detection of Glucose. ACS Omega.

[17] Buzzin A., Asquini R., Caputo D., de Cesare G. (2021). On-glass integrated su-8 waveguide and amorphous silicon photosensor for on-chip detection of biomolecules: Feasibility study on hemoglobin sensing. Sensors.

[18] Buzzin A., Asquini R., Caputo D., de Cesare G. (2022). Evanescent waveguide lab-on-chip for optical biosensing in food quality control. Photonics Res..

[19] Shehadeh A., Evangelou A., Kechagia D., Tataridis P., Chatzilazarou A., Shehadeh F. (2020). Effect of ethanol, glycerol, glucose/fructose and tartaric acid on the refractive index of model aqueous solutions and wine samples. Food Chem..

[20] Yeh Y.L. (2008). Real-time measurement of glucose concentration and average refractive index using a laser interferometer. Opt. Lasers Eng..

[21] Buzzin A., Asquini R., Caputo D., De Cesare G. Optical Detection of Analytes through Evanescent Waves in Lab-on-Chip Devices. Proceedings of the 2021 44th International Convention on Information, Communication and Electronic Technology, MIPRO.

[22] Liu G., Tian Y., Kan Y. (2005). Fabrication of high-aspect-ratio microstructures using SU8 photoresist. Microsyst. Technol..

[23] Girault P., Lorrain N., Poffo L., Guendouz M., Lemaitre J., Carré C., Gadonna M., Bosc D., Vignaud G. (2015). Integrated polymer micro-ring resonators for optical sensing applications. J. Appl. Phys..

[24] Lorenz H., Despont M., Fahrni N., LaBianca N., Renaud P., Vettiger P. (1997). SU-8: A low-cost negative resist for MEMS. J. Micromech. Microeng..

[25] Wang Y., Yang M., Wei G., Hu R., Luo Z., Li G. (2014). Improved PLS regression based on SVM classification for rapid analysis of coal properties by near-infrared reflectance spectroscopy. Sens. Actuators, B Chem..

[26] Alam B., Ferraro A., Caputo R., Asquini R. (2022). Optical properties and far field radiation of periodic nanostructures fed by an optical waveguide for applications in fluorescence and Raman scattering. Opt. Quantum Electron..

[27] Bertsch A., Renaud P. (2015). Special issue: 15 years of SU8 as MEMS material. Micromachines.

[28] Vasylieva N., Marinesco S., Barbier D., Sabac A. (2015). Silicon/SU8 multi-electrode micro-needle for in vivo neurochemical monitoring. Biosens. Bioelectron..

[29] De Cesare G., Nascetti A., Caputo D. (2015). Amorphous silicon p-i-n structure acting as light and temperature sensor. Sensors.

[30] Almasri M., Xu B., Castracane J. (2006). Amorphous silicon two-color microbolometer for uncooled IR detection. IEEE Sens. J..

[31] Sreejith S., Ajayan J., Kollem S., Sivasankari B. (2022). A Comprehensive Review on Thin Film Amorphous Silicon Solar Cells. Silicon.

[32] Della Corte F.G., Rao S. (2013). Use of amorphous silicon for active photonic devices. IEEE Trans. Electron Devices.

[33] De Vita C., Klitis C., Codreanu N., Ferrari G., Sorel M., Melloni A., Morichetti F. Compact amorphous-silicon visible-light monitor integrated in silicon nitride waveguides. Proceedings of the IEEE International Conference on Group IV Photonics GFP.

[34] Street R.A., Winer K. (2000). Material Properties of Hydrogenated Amorphous Silicon. Handbook of Semiconductor Technology: Electronic Structure and Properties of Semiconductors.

[35] Zangheri M., Di Nardo F., Mirasoli M., Anfossi L., Nascetti A., Caputo D., De Cesare G., Guardigli M., Baggiani C., Roda A. (2016). Chemiluminescence lateral flow immunoassay cartridge with integrated amorphous silicon photosensors array for human serum albumin detection in urine samples. Anal. Bioanal. Chem..

[36] Nathan M., Levy O., Goldfarb I., Ruzin A. (2003). Monolithic coupling of a SU8 waveguide to a silicon photodiode. J. Appl. Phys..

[37] Maniyara R.A., Graham C., Paulillo B., Bi Y., Chen Y., Herranz G., Baker D.E., Mazumder P., Konstantatos G., Pruneri V. (2021). Highly transparent and conductive ITO substrates for near infrared applications. APL Mater..

[38] Kalaga P.S., Kumar D., Ang D.S., Tsakadze Z. (2020). Highly Transparent ITO/HfO2/ITO Device for Visible-Light Sensing. IEEE Access.

[39] König T.A.F., Ledin P.A., Kerszulis J., Mahmoud M.A., El-Sayed M.A., Reynolds J.R., Tsukruk V.V. (2014). Electrically tunable plasmonic behavior of nanocube-polymer nanomaterials induced by a redox-active electrochromic polymer. ACS Nano.

[40] Minami T. (2005). Transparent conducting oxide semiconductors for transparent electrodes. Semicond. Sci. Technol..

[41] Asquini R., Buzzin A., Caputo D., De Cesare G. (2018). Integrated Evanescent Waveguide Detector for Optical Sensing. IEEE Trans. Compon. Packag. Manuf. Technol..

[42] Yusmawati W.Y.W., Chuah H.P., Mahmood M.Y.W. (2007). Optical properties and sugar content determination of commercial carbonated drinks using surface plasmon resonance. Am. J. Appl. Sci..

[43] Maier S.A. (2007). Plasmonics: Fundamentals and Applications.

[44] Xhoxhi M., Dudia A., Ymeti A. (2015). Interferometric Evanescent Wave Biosensor Principles and Parameters. IOSR J. Appl. Phys..

[45] Wang W., Deng G., Hu Z., Chen K., Wu J. (2023). Sensitive Evanescence-Field Waveguide Interferometer for Aqueous Nitro-Explosive Sensing. Chemosensors.

[46] Alam B., Buzzin A., Grossi F., Caputo D., Cesare G.D., Asquini R. Optical Interferometer with On-Chip Amorphous Silicon Photodiode for Biosensing Applications. Proceedings of the 2022 IEEE Photonics Conference, IPC 2022—Proceedings.

